# Effect of Herbs and Spices on Acceptance, Preference, and Intake of Vegetables Among Rural Adolescents

**DOI:** 10.1093/nutrit/nuaf170

**Published:** 2026-05-26

**Authors:** Kathleen L Keller, Barbara J Rolls

**Affiliations:** Department of Nutritional Sciences, The Pennsylvania State University; Department of Food Science, The Pennsylvania State University; Department of Nutritional Sciences, The Pennsylvania State University

**Keywords:** behavior, pediatrics, vegetables, diet

## Abstract

**Objectives:**

To test whether herbs and spices improve acceptance, preference, and intake of vegetables among rural middle and high school students.

**Background:**

Vegetable intake among rural children and adolescents is inadequate. Novel strategies are needed to increase vegetable intake in this population.

**Methods:**

This school-based study was conducted in 3 phases. In phase 1, recipes were developed for 8 vegetables using uniquely formulated spice blends (eg, dill, cardamom, cumin). To evaluate recipe acceptance, students (Ns = 96-110; aged 14-18 y) rated liking (100-mm visual analog scales) and preference (forced choice) for plain (oil and salt) versus seasoned vegetables. Both plain and seasoned had similar amounts of oil and salt, but the seasoned also had specially formulated herb and spice blends. In phase 2, herb and spice blends were added to 8 vegetables served in the cafeteria and their intake was compared to plain controls. In phase 3, we tested whether 5 repeated exposures to 2 of the recipes from phase 2 increased intake.

**Results:**

In phase 1, students generally liked and preferred the seasoned vegetables over the plain versions. However, in phase 2, vegetable selection was low (8%-15%) and overall intake was greater for plain relative to seasoned vegetables. In phase 3, repeated exposure to the dill spice blend increased students’ willingness to try vegetables seasoned with this blend in the future.

**Conclusions:**

Although herbs and spices increased vegetable acceptance and preference among rural school children, this did not consistently translate into increased selection or intake in the cafeteria setting. Increasing exposure to herbs and spices prior to implementation and involving students in intervention planning may help overcome barriers to seasoned vegetable intake in this population.

## INTRODUCTION

The National School Lunch Program serves more than 30 million children annually, providing ∼33% of a child’s daily energy needs.^1^ Thus, it plays a major role in the dietary health of American youth and is an important target for meeting dietary shortfalls. Following the implementation of the Healthy Hunger Free Kids Act of 2010,^2^ schools were required to offer a larger variety of vegetables as part of lunch.^3^ These policy changes improved dietary quality^4^ and reduced obesity^5^ among United States school children, but vegetable intake remains insufficient.^6^ Low liking of vegetables continues to be a barrier to increasing children’s consumption.^7^ Herbs and spices offer a promising strategy to enhance vegetable flavor without adding fat, sugar, and salt. These interventions may be particularly valuable in rural settings, where chronic health conditions such as obesity are 26% higher than in urban areas.^8^ In light of these public health challenges, from 2015-2017, we conducted a multiphase study to determine whether herbs and spices could improve vegetable preference, liking, and intake among rural school children.

## PHASE 1

### Methods

We collaborated with a local, combined middle/high school in rural, central Pennsylvania to complete the studies. The school was composed of ∼950 students (grades 6-12) who were predominantly White (96.3%) with ∼39% of students receiving free or reduce lunch (Student Poverty Concentration-2014). In phase 1 of the study (spring 2015), school food service staff, students, and parents at a rural high school participated. Sixty-two (*n* = 62) high school students enrolled in home economics classes volunteered to take surveys home to their parents, and 60 of those surveys were returned (∼97%). In addition, all food service workers (*n* = 13) completed surveys on the perceived barriers to vegetable preparation and consumption.

In addition to the surveys, we worked with professional research chefs at the McCormick Science Institute to develop 9 recipes that were evaluated by student focus groups (*n* = 33 students total). During these focus groups, students were asked current opinions on school vegetable preparations and preferred seasonings for raw and cooked vegetables. The students also tasted and discussed the new recipes. Based on these focus groups, the research chefs developed herb and spice blends for 6 cooked vegetables (ie, carrots, green beans, black beans and corn, cauliflower, broccoli, and sweet potatoes) and an herb and spice dip served with raw carrots.

### Taste Tests

To determine whether students liked the new vegetable recipes, we carried out a series of taste tests with high school students during the school lunch period. Approximately 100 students (14-18 years old) participated in each taste test where the seasoned vegetables were compared to plain controls. Both the control and seasoned vegetables were prepared with similar amounts of soybean oil and salt, but the seasoned also included herb and spice blends. A single vegetable was evaluated each day. Students were briefly informed about the taste tests and told their participation was voluntary. Those who chose to participate came to a table set up in the center of the cafeteria where they were provided with plate, fork, water, vegetable samples, test form, and pen. Instructions were provided on the form, and researchers were available should the students have questions. Students rated each vegetable on 100-mm unstructured line scales with ends labeled “the worst” and “the best.” Students were also asked which sample they preferred.

### Results

**Figure 1.**
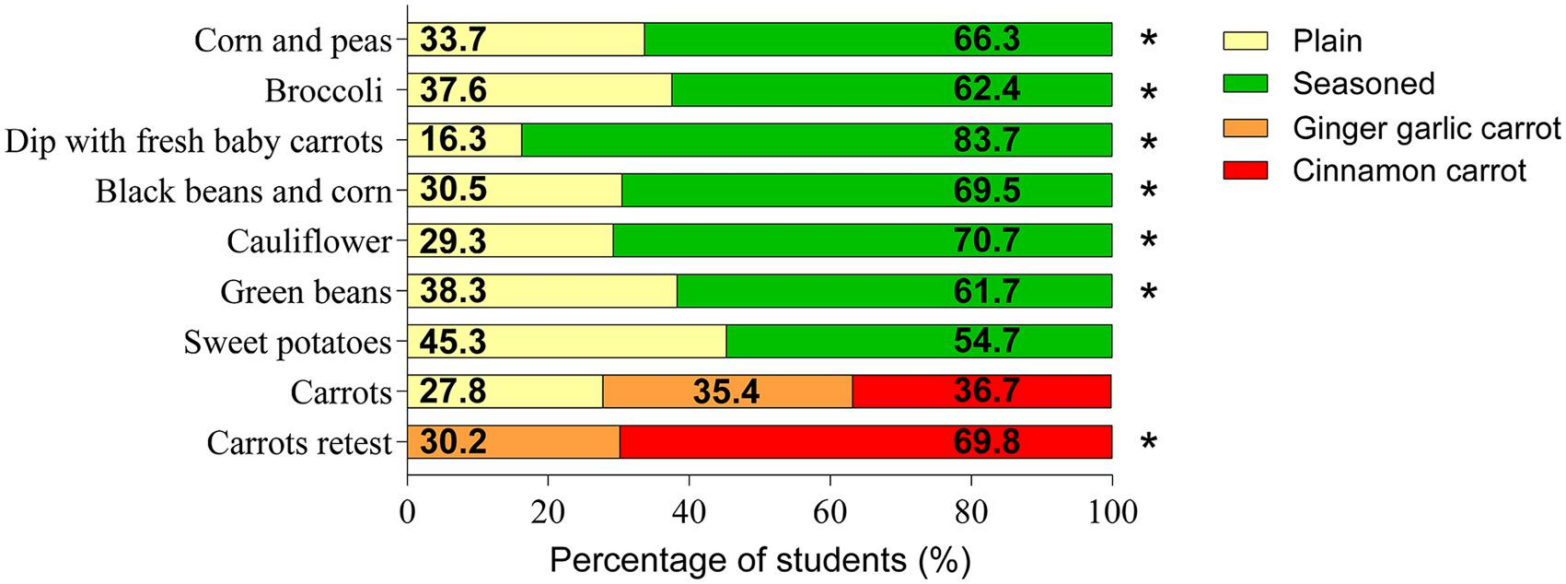
Percent of Students who Preferred Each Vegetable (Plain vs. Seasoned). Students were asked “which sample do you prefer?” χ^2^ tests were performed for each vegetable and an asterisk is used to denote significance at *P* < –.05).

Results of this first study showed that rural students reported limited exposure to herbs and spices before the study. Of the 20 herbs and spices asked about on surveys, only 9 (cinnamon, garlic powder, black pepper, chili powder, oregano, basil, parsley, onion powder, and paprika) were familiar to greater than 50% of the students. Despite this, the vegetables seasoned with herbs and spices were generally liked and preferred over plain versions of vegetables. Students liked the seasoned carrots and dip, black beans and corn, cauliflower, and broccoli over the plain versions, and cinnamon carrots were liked slightly more than Asian carrots. Similarly, when asked to choose a favorite, the seasoned recipes were preferred to the plain for all vegetables except sweet potatoes and carrots.^9^ As neither the ginger garlic or cinnamon carrot was preferred compared to plain carrots, a retest was conducted comparing the 2 seasoned carrots to each other. The retest showed that the cinnamon carrot was preferred to the ginger garlic carrot (Figure 1).

## PHASE 2

Based on the promising findings in the initial study, we carried out a follow-up study in spring and fall of 2017. The intervention compared students’ selection, intake, and reported willingness to eat 8 lightly salted (ie, “plain”) and 8 seasoned vegetables. The order in which students were presented with each vegetable condition (plain vs seasoned) was randomized. Before each testing day, blends of herbs and spices and the fresh dips were prepared in the laboratory at The Pennsylvania State University and transported to the school before lunch so that vegetables could be mixed with the spice blends and portioned by research staff in the cafeteria. Vegetables were preweighed and labels were provided for vegetables so students knew what they were selecting (ie, “new plain vegetable” or “new seasoned vegetable”). Following consumption, researchers postweighed the vegetables before they were deposited into the trash can.

**Figure 2.**
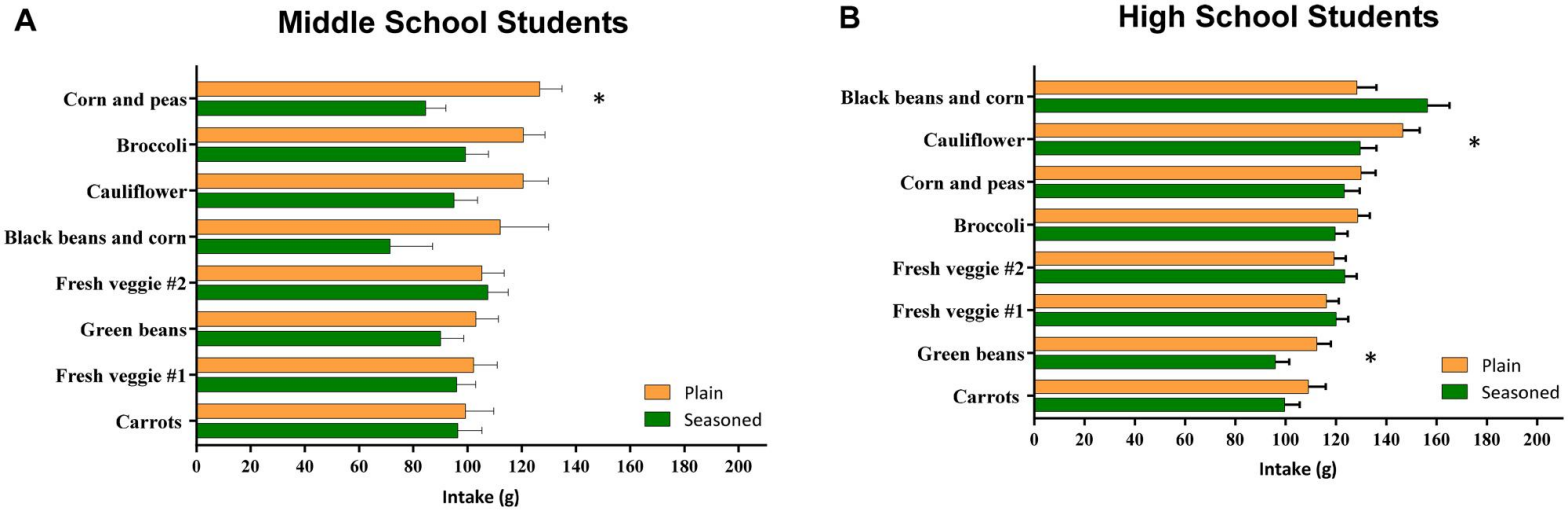
(A) Middle school students consumed more plain corn and peas than seasoned (*P *< .05). (B) High school students consumed more plain cauliflower and green beans than seasoned (*P *< .05).

On the lunch line, students were typically provided with 2 to 3 fruit options (eg, canned fruit, fruit juice) and 2 to 3 vegetable options (eg, fresh vegetable, steamed vegetable, French fried potatoes), with the intervention vegetable as 1 of these choices. Based on National School Lunch Regulations of Offer vs. Serve, students only had to take 1 vegetable or fruit option. As result, of 625 to 660 students served daily, only 6.6% to 17.6% chose the intervention vegetables. In addition, middle school students selected fewer vegetables overall than high school students; thus, data were separately analyzed in the 2 age groups. Overall, there were few significant differences, but for vegetables that differed by condition, students tended to eat more of the plain versions than the seasoned; however, high schoolers generally consumed more of the seasoned vegetables than middle schoolers (Figure 2).

## PHASE 3

Given the importance of repeated exposure to vegetables to increase liking,^10^ we conducted a third phase of the study to test the influence of multiple exposures to 2 recipes that were used in phase 2: Dillicious Broccoli and Fiesta Black Beans and Corn. These recipes were offered across the fall 2017 semester as part of the regular rotation of foods. Baseline intake, selection, and willingness to eat again were measured for each recipe twice during the preexposure and twice during the postexposure periods. Between collection dates, the seasoning blends for the Dillicious Broccoli blend and Fiesta Black Beans and Corn were added on 5 separate occasions to vegetables served during lunch. In this phase, the herb and spice blends were served on a variety of vegetables, in addition to broccoli and black beans and corn, to prevent the target vegetables from becoming monotonous. After offering the herb and spice blends for the semester, we saw an increases in the percentage of students who reported that they would eat the Dillicious Broccoli if it were served again (82.1% preexposure to 93.9% postexposure). For Fiesta Black Beans and Corn, 66.7% said they *would eat it again* preexposure and 92.3% reported they *would eat it again* postexposure, although this change was not statistically significant due to low overall selection of this vegetable both pre- and postexposure.

## DISCUSSION

These studies revealed that increased liking does not always translate to increased selection and intake, particularly in the school cafeteria. Although rural middle/high schoolers with limited exposure to herbs and spices reported liking and preferring seasoned vegetables over plain, when these vegetables were added to the cafeteria line, most students selected and ate more of the plain version. In hindsight, this result occurred because students had many competing fruits and vegetables to select from, and for students who selected the intervention vegetable, they opted for the familiar (plain) version that they were accustomed to over a novel seasoned recipe to which they had no or limited exposure. That middle schoolers were even less likely to select the seasoned versions than high schoolers suggested that exposure and familiarity with the seasonings was a barrier to selection. This conclusion was further confirmed by our follow-up study in which semester-long exposure to 2 spice blends increased willingness to consume again.

The promising outcomes we found in our initial acceptance study support a potential role for the use of herbs and spices to increase vegetable acceptance among rural middle and high schoolers. The beneficial health properties of herbs and spices combined with their ability to improve the flavor of school lunch vegetables without added sugar, salt, and fat provide opportunities to continue to develop healthy and flavorful school lunches. Future studies are needed in rural children to identify additional barriers to integrating novel herbs and spices into the diet. For example, to increase student’s exposure to novel herb and spice blends, food service staff could offer “free samples” to students to taste before they are incorporated into the cafeteria line. Student ambassadors could be enrolled to help implement the intervention and could serve as role models to encourage other students to try the new recipes. Novel herb and spice blends could also be added to condiment stations located in the cafeteria, which might encourage some students to try new flavors. Finally, working with cafeteria staff in menu planning to limit some of the highly palatable competing options could encourage students to try novel preparations of vegetables at school.

## Supplementary Material

nuaf170_Supplementary_Data

## Data Availability

Data described in the manuscript can be requested from the corresponding author.

## References

[1] Cullen KW , Chen TA. The contribution of the USDA school breakfast and lunch program meals to student daily dietary intake. Prev Med Rep. 2017;5:82-85. 27957411 10.1016/j.pmedr.2016.11.016 PMC5149064

[2] Healthy, Hunger-Free Kids Act of 2010. Pub L No. 111-296, 124 Stat 3183. 2010.

[3] Nutrition Standards in the National School Lunch and School Breakfast Programs. Final Rule. Fed Regist. 2012;77:4088-4167. 22359796

[4] Kinderknecht K , Harris C, Jones-Smith J. Association of the Healthy, Hunger-Free Kids Act with dietary quality among children in the US National School Lunch Program. JAMA. 2020;324:359-368. 32721008 10.1001/jama.2020.9517 PMC7388023

[5] Kenney EL , Barrett JL, Bleich SN, et al Impact of The Healthy, Hunger-Free Kids Act on obesity trends. Health Affairs. 2020;39:1122-1129. 32634356 10.1377/hlthaff.2020.00133 PMC7961790

[6] U.S. Department of Agriculture; U.S. Department of Health and Human Services. *Dietary Guidelines for Americans, 2020-2025*. 9th ed. 2020. Accessed May 15th, 2025. https://www.dietaryguidelines.gov

[7] Appleton KM , Hemingway A, Saulais L, et al Increasing vegetable intakes: Rationale and systematic review of published interventions. Eur J Nutr. 2016;55:869-896. 26754302 10.1007/s00394-015-1130-8 PMC4819941

[8] Johnson JA , Johnson AM. Urban-rural differences in childhood and adolescent obesity in the United States: a systematic review and meta-analysis. Child Obes. 2015;11:233-241. 25928227 10.1089/chi.2014.0085

[9] Fritts JR , Fort C, Quinn Corr A, et al Herbs and spices increase liking and preference for vegetables among rural high school students. Food Qual Preference. 2018;68:125-134.

[10] Spill MK , Johns K, Callahan EH, et al Repeated exposure to food and food acceptability in infants and toddlers: a systematic review. Am J Clin Nutr. 2019;109:978S-989S. 30982874 10.1093/ajcn/nqy308

