# Supplementary material for: Effect of Herbs and Spices on Acceptance, Preference, and Intake of Vegetables Among Rural Adolescents
Source: Nutr Rev. 2026 May 26;84(Suppl 1):117–20. doi: 10.1093/nutrit/nuaf170 (PMC13201869; doi:10.1093/nutrit/nuaf170)
Supplement: nuaf170_Supplementary_Data [file nuaf170_supplementary_data.docx]

**Supplementary Material**

Supporting table S1: PRISMA checklist

Supporting table 2: Search strategy

Supporting table 3: Summary of evidence table for conditional cash transfers

Supporting table 4: Summary of evidence table for unconditional cash transfers

Supporting table 5: Summary of evidence table for food vouchers

**Supporting table 1: PRISMA checklist**

| **Section and Topic** | **Item #** | **Checklist item** | **Location where item is reported** |
| --- | --- | --- | --- |
| **TITLE** | | |  |
| Title | 1 | Identify the report as a systematic review. | Page 1 |
| **ABSTRACT** | | |  |
| Abstract | 2 | See the PRISMA 2020 for Abstracts checklist. | Page 2 |
| **INTRODUCTION** | | |  |
| Rationale | 3 | Describe the rationale for the review in the context of existing knowledge. | Page 4-5 |
| Objectives | 4 | Provide an explicit statement of the objective(s) or question(s) the review addresses. | Page 5 |
| **METHODS** | | |  |
| Eligibility criteria | 5 | Specify the inclusion and exclusion criteria for the review and how studies were grouped for the syntheses. | Page 5-6 and Table 1 |
| Information sources | 6 | Specify all databases, registers, websites, organisations, reference lists and other sources searched or consulted to identify studies. Specify the date when each source was last searched or consulted. | Page 6-7 |
| Search strategy | 7 | Present the full search strategies for all databases, registers and websites, including any filters and limits used. | Table S2 |
| Selection process | 8 | Specify the methods used to decide whether a study met the inclusion criteria of the review, including how many reviewers screened each record and each report retrieved, whether they worked independently, and if applicable, details of automation tools used in the process. | Page 6-7 |
| Data collection process | 9 | Specify the methods used to collect data from reports, including how many reviewers collected data from each report, whether they worked independently, any processes for obtaining or confirming data from study investigators, and if applicable, details of automation tools used in the process. | Page 7 |
| Data items | 10a | List and define all outcomes for which data were sought. Specify whether all results that were compatible with each outcome domain in each study were sought (e.g. for all measures, time points, analyses), and if not, the methods used to decide which results to collect. | Page 6 |
|  | 10b | List and define all other variables for which data were sought (e.g. participant and intervention characteristics, funding sources). Describe any assumptions made about any missing or unclear information. | Page 7 |
| Study risk of bias assessment | 11 | Specify the methods used to assess risk of bias in the included studies, including details of the tool(s) used, how many reviewers assessed each study and whether they worked independently, and if applicable, details of automation tools used in the process. | Page 8 |
| Effect measures | 12 | Specify for each outcome the effect measure(s) (e.g. risk ratio, mean difference) used in the synthesis or presentation of results. | Page 7-8 |
| Synthesis methods | 13a | Describe the processes used to decide which studies were eligible for each synthesis (e.g. tabulating the study intervention characteristics and comparing against the planned groups for each synthesis (item #5)). | Page 7-8 |
|  | 13b | Describe any methods required to prepare the data for presentation or synthesis, such as handling of missing summary statistics, or data conversions. | Page 7-8 |
|  | 13c | Describe any methods used to tabulate or visually display results of individual studies and syntheses. | Page 7-8 |
|  | 13d | Describe any methods used to synthesize results and provide a rationale for the choice(s). If meta-analysis was performed, describe the model(s), method(s) to identify the presence and extent of statistical heterogeneity, and software package(s) used. | Page 6-8 |
|  | 13e | Describe any methods used to explore possible causes of heterogeneity among study results (e.g. subgroup analysis, meta-regression). | Page 7-8 |
|  | 13f | Describe any sensitivity analyses conducted to assess robustness of the synthesized results. | Page 7-8 |
| Reporting bias assessment | 14 | Describe any methods used to assess risk of bias due to missing results in a synthesis (arising from reporting biases). | Page 8 |
| Certainty assessment | 15 | Describe any methods used to assess certainty (or confidence) in the body of evidence for an outcome. | Page 8 |
| **RESULTS** | | |  |
| Study selection | 16a | Describe the results of the search and selection process, from the number of records identified in the search to the number of studies included in the review, ideally using a flow diagram. | Page 8 and Figure 1 |
|  | 16b | Cite studies that might appear to meet the inclusion criteria, but which were excluded, and explain why they were excluded. | Page 8 |
| Study characteristics | 17 | Cite each included study and present its characteristics. | Page 9 and Table 2 |
| Risk of bias in studies | 18 | Present assessments of risk of bias for each included study. | Page 10 and Figure 2 |
| Results of individual studies | 19 | For all outcomes, present, for each study: (a) summary statistics for each group (where appropriate) and (b) an effect estimate and its precision (e.g. confidence/credible interval), ideally using structured tables or plots. | Page 10-11 and Tables S3 – S5 |
| Results of syntheses | 20a | For each synthesis, briefly summarise the characteristics and risk of bias among contributing studies. | Page 10-11 and Tables S3 – S5 |
|  | 20b | Present results of all statistical syntheses conducted. If meta-analysis was done, present for each the summary estimate and its precision (e.g. confidence/credible interval) and measures of statistical heterogeneity. If comparing groups, describe the direction of the effect. | Page 10-11 and Tables S3 – S5 |
|  | 20c | Present results of all investigations of possible causes of heterogeneity among study results. | Page 10-11 and Tables S3 – S5 |
|  | 20d | Present results of all sensitivity analyses conducted to assess the robustness of the synthesized results. | Page 10-11 and Tables S3 – S5 |
| Reporting biases | 21 | Present assessments of risk of bias due to missing results (arising from reporting biases) for each synthesis assessed. | Page 10-11 and Tables S3 – S5 |
| Certainty of evidence | 22 | Present assessments of certainty (or confidence) in the body of evidence for each outcome assessed. | Page 10-11 and Tables S3 – S5 |
| **DISCUSSION** | | |  |
| Discussion | 23a | Provide a general interpretation of the results in the context of other evidence. | Page 12-14 |
|  | 23b | Discuss any limitations of the evidence included in the review. | Page 12-14 |
|  | 23c | Discuss any limitations of the review processes used. | Page 12-14 |
|  | 23d | Discuss implications of the results for practice, policy, and future research. | Page 12-14 |
| **OTHER INFORMATION** | | |  |
| Registration and protocol | 24a | Provide registration information for the review, including register name and registration number, or state that the review was not registered. | Page 3 |
|  | 24b | Indicate where the review protocol can be accessed, or state that a protocol was not prepared. | Page 3 and Page 5 |
|  | 24c | Describe and explain any amendments to information provided at registration or in the protocol. | Page 14 |
| Support | 25 | Describe sources of financial or non-financial support for the review, and the role of the funders or sponsors in the review. | Page 15 |
| Competing interests | 26 | Declare any competing interests of review authors. | Page 15 |
| Availability of data, code and other materials | 27 | Report which of the following are publicly available and where they can be found: template data collection forms; data extracted from included studies; data used for all analyses; analytic code; any other materials used in the review. | All data presented in the manuscript and supplementary table. |

**Supporting table S2: Search strategy**

MEDLINE

1 exp infant/ or exp child, preschool/ or (infant* or toddler* or baby or babies or preschool or newborn* or neonate* or kindergarten or under-5* or "under 5*" or under-five or "under five" or kid or paediatr* or pediatr* or child*).mp.

AND

2 exp malnutrition/ or exp infant nutrition disorders/ or exp protein-energy malnutrition/ or exp wasting syndrome/ or (malnutrition or malnourish* or undernutrition or undernourish* or wasting or wasted).mp.

AND

3 exp diet/ or exp food/ or exp infant food/ or exp food, fortified/ or exp food, formulated/ or exp dietary supplements/ or exp food assistance/ or exp energy intake/ or exp dietary proteins/ or exp dietary fat/ or exp counseling/ or exp education/ or exp prenatal care/ or exp breast feeding/ or exp weaning/

OR

(diet* adj4 diversity or diet* adj4 quality or diet* adj4 variety or food* adj4 variety or nutri* adj4 diversity or nutri* adj4 adequacy or diet* adj4 pattern* or nutri* adj4 pattern* or "food assistance" or "food distribution" or "food aid" or "food program*" or supplement* or micronutrient* or "lipid-based nutrient supplement*" or “lipid nutrient supplement*” or blend* or nutributter or counselling or counseling or intervention* or strateg* or education* or "social protection" or "cash transfer*" or "cash incentive*" or voucher* or CCT or "income supplement*" or "social welfare" or "social polic*" or "food ration*" or "food basket*" or "breast feed*" or breastfeed* or "breast fed" or breastfed or EBF or "human milk" or “breast milk” or colostrum or "milk bank" or "feeding bank" or IYCF or "infant and young child feeding" or “young child feed*” or "infant feed*" or "complementary feed*" or "supplement* feed*" or "complementary food*" or "supplement* food*" or wean* or "infant food*" or "young child food*").mp.

Ovid MEDLINE: Epub Ahead of Print, In-Process & Other Non-Indexed Citations, Ovid MEDLINE® Daily and Ovid MEDLINE® <1946-Present>

1 exp infant/ or exp child, preschool/ or (infant* or toddler* or baby or babies or preschool* or newborn* or neonate* or kindergarten or under-5* or "under 5*" or under-five or "under five" or kid* or paediatr* or pediatr* or child*).mp.

2 exp malnutrition/ or exp infant nutrition disorders/ or exp protein-energy malnutrition/ or exp wasting syndrome/ or (malnutrition or malnourish* or undernutrition or undernourish* or wasting or wasted).mp.

3 exp diet/ or exp food/ or exp infant food/ or exp food, fortified/ or exp food, formulated/ or exp dietary supplements/ or exp food assistance/ or exp energy intake/ or exp dietary proteins/ or exp dietary fat/ or exp counseling/ or exp education/ or exp prenatal care/ or exp breast feeding/ or exp weaning/

4 ("dietary diversity" or "diet diversity" or "diet quality" or "dietary quality" or "dietary quality index" or "dietary variety" or "diet variety" or "dietary diversity score" or "food variety score" or "nutritional diversity" or "nutrient diversity" or "nutritional functional diversity" or "nutritional adequacy" or "nutrient adequacy" or "nutrition adequacy" or "dietary pattern*" or "diet pattern*" or "nutritional pattern*" or "nutrition pattern*" or "food assistance" or "food distribution" or "food aid" or "nutrition assistance" or "food program*" or supplement* or micronutrient* or "lipid-based nutrient supplement*" or "lipid nutrient supplement" or blend* or nutributter or counselling or counseling or intervention* or strateg* or education* or "social protection" or "cash transfer*" or "cash incentive*" or CCT or "income supplement*" or "social welfare" or "social polic*" or "food ration*" or "food basket*" or voucher* or "breast feed*" or breastfeed* or "breast fed" or breastfed or "human milk" or "breast milk" or colostrum or "milk bank" or "feeding bank" or IYCF or "infant and young child feeding" or "young child feed*" or "infant feed*" or "complementary feed*" or "supplement* feed*" or "complementary food*" or "supplement* food*" or wean* or "infant food*" or "young child food*").mp.

5 3 or 4

6 1 and 2 and 5

7 limit 6 to yr="2021 -Current"

**Supporting table 3: Summary of evidence table for conditional cash transfers**

| **Certainty assessment** | | | | | | | **№ of patients** | | **Effect** | | **Certainty** | **Importance** |
| --- | --- | --- | --- | --- | --- | --- | --- | --- | --- | --- | --- | --- |
| **№ of studies** | **Study design** | **Risk of bias** | **Inconsistency** | **Indirectness** | **Imprecision** | **Other considerations** | **conditional cash transfers** | **control** | **Relative (95% CI)** | **Absolute (95% CI)** |  |  |
| **Prevalence of wasting** | | | | | | | | | | | | |
| 3 | randomised trials | serious^a^ | not serious | serious^b^ | serious^c^ | none | 0/0 | 281/1481 (19.0%) | **OR 0.97** (0.91 to 1.02) | **5 fewer per 1,000** (from 14 fewer to 3 more) | ⨁◯◯◯ Very low | CRITICAL |
| **Prevalence of severe wasting** | | | | | | | | | | | | |
| 1 | randomised trials | serious^d^ | not serious | not serious | not serious^e^ | none | 0/0 | 126/1394 (9.0%) | **OR 0.97** (0.94 to 1.00) | **2 fewer per 1,000** (from 5 fewer to 0 fewer) | ⨁⨁⨁◯ Moderate | CRITICAL |
| **WHZ** | | | | | | | | | | | | |
| 1 | randomised trials | not serious | not serious | not serious | extremely serious^f^ | none | 126 | 124 | - | MD **0.55 lower** (0.86 lower to 0.24 lower) | ⨁◯◯◯ Very low | IMPORTANT |
| **WAZ** | | | | | | | | | | | | |
| 1 | randomised trials | not serious | not serious | not serious | extremely serious^g^ | none | 126 | 124 | - | MD **0.08 lower** (0.31 lower to 0.15 higher) | ⨁◯◯◯ Very low | IMPORTANT |
| **Prevalence of underweight (WAZ <-2)** | | | | | | | | | | | | |
| 2 | randomised trials | serious^h^ | not serious | serious^b^ | serious^i^ | none | 53/605 (8.8%) | 90/681 (13.2%) | **RR 0.68** (0.49 to 0.93) | **42 fewer per 1,000** (from 67 fewer to 9 fewer) | ⨁◯◯◯ Very low | IMPORTANT |

CI: confidence interval; MD: mean difference; OR: odds ratio; RR: risk ratio

**Explanations**

a. Serious risk of bias: One study with overall high risk of bias contributes the vast majority of the information.

b. Serious indirectness: Population of interest of included trials indirect to target guideline population

c. Serious imprecision: The 95% CIs around the absolute effect does cross the null and includes potentially meaningful benefits to trivial harms using a population perspective. Control event rate imputed from Kusuma 2017 which contributed the vast majority of the information.

d. Serious risk of bias: One study judged as having overall high risk of bias.

e. No imprecision: The 95% CI around the absolute effect ranges from trivial benefit to no effect using a population perspective. Control event rate imputed from Kusuma 2017

f. Extremely serious imprecision: The 95% CIs around the absolute effect does not cross the null threshold but includes an extremely wide absolute confidence interval. Additionally this is a large effect and the optimal information size is not met (<400 participants).

g. Extremely serious imprecision: The 95% CIs around the absolute effect crosses the null threshold and includes a potentially large harms and meaningful benefit. Additionally the optimal information size is not met (<400 participants).

h. Serious risk of bias: One study with overall high risk of bias (Maluccio 2004) contributes the majority of the information.

i. Serious imprecision: The 95% CIs around the absolute effect does not cross the null threshold but includes potentially large to trivial benefit using a population perspective

**Supporting table 4: Summary of evidence table for unconditional cash transfers**

| **Certainty assessment** | | | | | | | **№ of patients** | | **Effect** | | **Certainty** | **Importance** |
| --- | --- | --- | --- | --- | --- | --- | --- | --- | --- | --- | --- | --- |
| **№ of studies** | **Study design** | **Risk of bias** | **Inconsistency** | **Indirectness** | **Imprecision** | **Other considerations** | **unconditional cash transfers** | **control** | **Relative (95% CI)** | **Absolute (95% CI)** |  |  |
| **Prevalence of wasting (Fenn 2017, standard cash intervention arm only)** | | | | | | | | | | | | |
| 1 | randomised trials | serious^a^ | not serious | not serious | very serious^b^ | none | 0/0 | 184/840 (21.9%) | **OR 1.09** (0.64 to 1.86) | **15 more per 1,000** (from 67 fewer to 124 more) | ⨁◯◯◯ Very low | CRITICAL |
| **Prevalence of wasting (Fenn 2017, double cash intervention arm only)** | | | | | | | | | | | | |
| 1 | randomised trials | serious^a^ | not serious | not serious | serious^c^ | none | 0/0 | 184/840 (21.9%) | **OR 0.52** (0.29 to 0.93) | **92 fewer per 1,000** (from 144 fewer to 12 fewer) | ⨁⨁◯◯ Low | CRITICAL |
| **Prevalence of severe wasting (Fenn 2017, standard cash intervention arm only)** | | | | | | | | | | | | |
| 1 | randomised trials | serious^a^ | not serious | not serious | very serious^d^ | none |  | 62/852 (7.3%) | **OR 0.98** (0.38 to 2.53) | **1 fewer per 1,000** (from 44 fewer to 93 more) | ⨁◯◯◯ Very low | CRITICAL |
| **Prevalence of severe wasting (Fenn 2017, double cash intervention arm only)** | | | | | | | | | | | | |
| 1 | randomised trials | serious^a^ | not serious | not serious | serious^e^ | none | 0/0 | 62/852 (7.3%) | **OR 0.37** (0.13 to 1.05) | **45 fewer per 1,000** (from 63 fewer to 3 more) | ⨁⨁◯◯ Low | CRITICAL |
| **Incidence of wasting** | | | | | | | | | | | | |
| 1 | randomised trials | not serious | not serious | not serious | very serious^f^ | none | 0/0 | 0/0 | **RR 0.92** (0.64 to 1.32) | **1 fewer per 1,000** (from 1 fewer to 1 fewer) | ⨁⨁◯◯ Low | CRITICAL |
| **Cumulative incidence of wasting** | | | | | | | | | | | | |
| 1 | randomised trials | not serious | not serious | not serious | very serious^f^ | none | 0/0 | 0/0 | **RR 0.97** (0.83 to 1.15) | **1 fewer per 1,000** (from 1 fewer to 1 fewer) | ⨁⨁◯◯ Low | CRITICAL |
| **WHZ** | | | | | | | | | | | | |
| 1 | randomised trials | not serious | not serious | not serious | serious^g^ | none | 630 | 620 | - | MD **0.05 higher** (0.05 lower to 0.15 higher) | ⨁⨁⨁◯ Moderate | IMPORTANT |
| **MUAC (cm)** | | | | | | | | | | | | |
| 1 | randomised trials | not serious | not serious | not serious | serious^h^ | none | 630 | 620 | - | MD **0.01 higher** (0.11 lower to 0.13 higher) | ⨁⨁⨁◯ Moderate | IMPORTANT |
| **Incidence of diarrhea** | | | | | | | | | | | | |
| 1 | randomised trials | serious^a^ | not serious | not serious | not serious | none | 0/0 | 0/0 | **Rate ratio 1.00** (0.97 to 1.03) | **-- per 1000 patient(s) per years**  (from -- to --) | ⨁⨁⨁◯ Moderate | IMPORTANT |
| **Prevalence of diarrhea (standard cash)** | | | | | | | | | | | | |
| 1 | randomised trials | serious^a^ | not serious | not serious | serious^i^ | none |  |  | **OR 1.05** (0.67 to 1.64) | **1 fewer per 1,000** (from 2 fewer to 1 fewer) | ⨁⨁◯◯ Low | IMPORTANT |
| **Prevalence of diarrhea (double cash)** | | | | | | | | | | | | |
| 1 | randomised trials | serious^a^ | not serious | not serious | serious^i^ | none | 0/0 | 0/0 | **OR 0.87** (0.55 to 1.37) | **1 fewer per 1,000** (from 1 fewer to 1 fewer) | ⨁⨁◯◯ Low | IMPORTANT |
| **Incidence of fever** | | | | | | | | | | | | |
| 1 | randomised trials | serious^a^ | not serious | not serious | not serious | none | 0/0 | 0/0 | **Rate ratio 0.98** (0.95 to 1.02) | **-- per 1000 patient(s) per years**  (from -- to --) | ⨁⨁⨁◯ Moderate | IMPORTANT |
| **Prevalence of fever/malaria (standard cash)** | | | | | | | | | | | | |
| 1 | randomised trials | serious^a^ | not serious | not serious | serious^j^ | none |  |  | **OR 0.64** (0.46 to 0.90) | **1 fewer per 1,000** (from 1 fewer to 0 fewer) | ⨁⨁◯◯ Low | IMPORTANT |
| **Prevalence of fever/malaria (double cash)** | | | | | | | | | | | | |
| 1 | randomised trials | serious^a^ | not serious | not serious | serious^j^ | none | 0/0 | 0/0 | **OR 0.63** (0.45 to 0.89) | **1 fewer per 1,000** (from 1 fewer to 0 fewer) | ⨁⨁◯◯ Low | IMPORTANT |
| **Incidence of respiratory tract infections** | | | | | | | | | | | | |
| 1 | randomised trials | serious^a^ | not serious | not serious | not serious | none | 0/0 | 0/0 | **Rate ratio 0.79** (0.78 to 0.81) | **-- per 1000 patient(s) per years**  (from -- to --) | ⨁⨁⨁◯ Moderate | IMPORTANT |
| **Prevalence of acute respiratory infection (standard cash)** | | | | | | | | | | | | |
| 1 | randomised trials | serious^a^ | not serious | not serious | serious^k^ | none |  |  | **OR 0.73** (0.51 to 1.04) | **1 fewer per 1,000** (from 1 fewer to 1 fewer) | ⨁⨁◯◯ Low | IMPORTANT |
| **Prevalence of acute respiratory infection (double cash)** | | | | | | | | | | | | |
| 1 | randomised trials | serious^a^ | not serious | not serious | serious^j^ | none | 0/0 | 0/0 | **OR 0.57** (0.40 to 0.81) | **1 fewer per 1,000** (from 1 fewer to 0 fewer) | ⨁⨁◯◯ Low | IMPORTANT |
| **Mortality** | | | | | | | | | | | | |
| 1 | randomised trials | not serious | not serious | not serious | very serious^l^ | none | 47/644 (7.3%) | 32/634 (5.0%) | **RR 1.45** (0.94 to 2.24) | **23 more per 1,000** (from 3 fewer to 63 more) | ⨁⨁◯◯ Low | IMPORTANT |
| **Mortality (IRR)** | | | | | | | | | | | | |
| 1 | randomised trials | serious^m^ | not serious | not serious | serious^n^ | none |  |  | **RR 0.97** (0.92 to 1.02) | **1 fewer per 1,000** (from 1 fewer to 1 fewer) | ⨁⨁◯◯ Low | IMPORTANT |

CI: confidence interval; MD: mean difference; OR: odds ratio; RR: risk ratio

**Explanations**

a. Serious risk of bias: One study judged as having overall high risk of bias.

b. Very serious imprecision: The 95% CIs around the absolute effect does cross the null and ranges from a potentially large benefits and harms using a population perspective.

c. Serious imprecision: The 95% CIs around the absolute effect does not cross the null but ranges from a potentially small to large benefit using a population perspective.

d. Very serious imprecision: The 95% CIs around the absolute effect does cross the null and ranges from a potentially meaningful benefits to large harms using a population perspective.

e. Serious imprecision: The 95% CIs around the absolute effect does cross the null and ranges from a trivial harm to potentially large benefit using a population perspective.

f. Very serious imprecision: The 95% CIs around the relative effect crosses the null and includes potential large harms and benefits. Absolute effect unavailable

g. Serious imprecision: The 95% CIs around the absolute effect crosses the null and includes potential meaningful harms and benefits using a population perspective.

h. Serious imprecision: The 95% CIs around the absolute effect crosses the null and includes potential meaningful harms and benefits using a population perspective.

i. Serious imprecision: The 95% CIs around the relative effect does cross the null and includes potentially meaningful benefits and harms. Absolute effects not available.

j. Serious imprecision: The 95% CIs around the relative effect does not cross the null but includes a wide range of appreciable benefits. Absolute effects not available.

k. Serious imprecision: The 95% CIs around the relative effect does cross the null and includes a potential meaningful benefits and trivial harms. Absolute effects not available.

l. Very serious imprecision: The 95% CIs around the absolute effect does cross the null and includes trivial benefit to potentially large harms using a population perspective.

m. Serious Risk of Bias: Unclear data points for this effect estimate. Data not shown in paper and request trialists for further clarity pending. [preliminary judgment]

n. Serious imprecision: The 95% CIs around the relative effect does cross the null and includes potentially meaningful benefits and trivial harms. Absolute effects not available

**Supporting table 5: Summary of evidence table for food vouchers**

| **Certainty assessment** | | | | | | | **№ of patients** | | **Effect** | | **Certainty** | **Importance** |
| --- | --- | --- | --- | --- | --- | --- | --- | --- | --- | --- | --- | --- |
| **№ of studies** | **Study design** | **Risk of bias** | **Inconsistency** | **Indirectness** | **Imprecision** | **Other considerations** | **food vouchers** | **control** | **Relative (95% CI)** | **Absolute (95% CI)** |  |  |
| **Prevalence of wasting** | | | | | | | | | | | | |
| 1 | randomised trials | serious^a^ | not serious | not serious | very serious^b^ | none | 0/0 | 184/840 (21.9%) | **OR 1.16** (0.67 to 2.01) | **26 more per 1,000** (from 61 fewer to 141 more) | ⨁◯◯◯ Very low | CRITICAL |
| **Prevalence of severe wasting** | | | | | | | | | | | | |
| 1 | randomised trials | serious^a^ | not serious | not serious | very serious^b^ | none | 0/0 | 62/852 (7.3%) | **OR 1.27** (0.45 to 3.55) | **18 more per 1,000** (from 39 fewer to 145 more) | ⨁◯◯◯ Very low | CRITICAL |
| **Prevalence of diarrhea** | | | | | | | | | | | | |
| 1 | randomised trials | serious^a^ | not serious | not serious | serious^c^ | none | 0/0 | 0/0 | **OR 0.99** (0.64 to 1.54) | **1 fewer per 1,000** (from 2 fewer to 1 fewer) | ⨁⨁◯◯ Low | IMPORTANT |
| **Prevalence of acute respiratory infection** | | | | | | | | | | | | |
| 1 | randomised trials | serious^a^ | not serious | not serious | serious^c^ | none | 0/0 | 0/0 | **OR 0.87** (0.61 to 1.24) | **1 fewer per 1,000** (from 1 fewer to 1 fewer) | ⨁⨁◯◯ Low | IMPORTANT |
| **Prevalence of fever/malaria** | | | | | | | | | | | | |
| 1 | randomised trials | serious^a^ | not serious | not serious | serious^c^ | none | 0/0 | 0/0 | **OR 0.87** (0.62 to 1.22) | **1 fewer per 1,000** (from 1 fewer to 1 fewer) | ⨁⨁◯◯ Low | IMPORTANT |

CI: confidence interval; OR: odds ratio

**Explanations**

a. Serious risk of bias: One study of high overall risk of bias (Fenn 2017).

b. Very serious imprecision: The 95% CIs around the absolute effect crosses the null and includes large benefits and very large harms using a population perspective.

c. Serious imprecision: The 95% CIs around the relative effect does cross the null and includes potentially meaningful benefits and harms using a population perspective. Absolute effects not available.
